# The Link Between Energy-Related Sensations and Metabolism: Implications for Treating Fatigue

**DOI:** 10.3389/fpsyg.2022.920556

**Published:** 2022-06-21

**Authors:** Marco Filippi, Rainer Krähenmann, Patrick Fissler

**Affiliations:** ^1^Psychiatric Services Thurgau, Münsterlingen, Switzerland; ^2^University Hospital for Psychiatry and Psychotherapy, Paracelsus Medical University, Salzburg, Austria; ^3^Department of Psychiatry, Psychotherapy and Psychosomatics, University of Zürich, Zürich, Switzerland

**Keywords:** fatigue, energy metabolism, mitochondria, autonomic nervous system, inflammation, energy sensations

## Abstract

Energy-related sensations include sensation of energy and fatigue as well as subjective energizability and fatigability. First, we introduce interdisciplinary useful definitions of all constructs and review findings regarding the question of whether sensations of fatigue and energy are two separate constructs or two ends of a single dimension. Second, we describe different components of the bodily energy metabolism system (e.g., mitochondria; autonomic nervous system). Third, we review the link between sensation of fatigue and different components of energy metabolism. Finally, we present an overview of different treatments shown to affect both energy-related sensations and metabolism before outlining future research perspectives.

## Defining Different Kinds of Energy-Related Sensations

*Energy-related sensations* such as fatigue are phenomena that, despite being researched in various fields, such as medicine, cognitive psychology, or exercise physiology, still lack consensus definitions and taxonomy (Pattyn et al., 2018; Skau et al., 2021). As Pattyn et al. (2018) and Skau et al. (2021) point out, this fragmentation of knowledge hinders innovation, interdisciplinary collaboration, communication, and can lead to confusion or to a situation where advances in one field do not reach other fields, causing the phenomenon of “reinventing the wheel.” Skau et al. (2021) have proposed two criteria that an interdisciplinary definition needs to fulfil: the first is non-circularity, meaning that no part of the term defined (in this case fatigue) should be defined by itself or have already been used in the definitions of a prior definition. An example of circularity would be defining fatigue as “feeling of exhaustion or lack of energy,” when exhaustion itself was defined as “feeling of fatigue or lack of energy.” The second criterion is finiteness, according to which at the end of the definition chain there should be some primitives or undefined terms that get their meaning from meta-linguistic practices, such as ostensive procedures, i.e., pointing out examples, such as pointing to a blue object to define the colour blue (Skau et al., 2021). In addition to these two criteria, Skau et al. (2021) propose four desiderata: broadness (the definition is broad enough so that it can be used in different fields), precision (the definition is precise enough to avoid multiple interpretations), neutrality (the definition should not depend on a certain theory), and phenomenon focus (the definition should not involve explanations or should do so to a minimal extent).

### Defining Sensation of Fatigue and Energy

In this section, we propose a definition of fatigue and related constructs, after introducing recently proposed definitions which serve as the basis for our proposal. Based on the two criteria and four desiderata, Skau et al. (2021) formulate a definition of the *sensation of fatigue* as “if and only if there is a sensation of (i) feeling the need for rest, or (ii) mismatch between effort expended and actual performance” (Skau et al., 2021, p. 3). This sensation of fatigue can then encompass different domains, such as cognitive, physical, or emotional (Skau et al., 2021). Among the numerous instruments that assess multiple dimensions of fatigue, the most common domains are *cognitive*, *physical*, and *emotional* (Whitehead, 2009). Some instruments also include a *motivational* component, such as the Multidimensional Fatigue Inventory (MFI; Smets et al., 1995), and a *social* component, such as the Fatigue Impact Scale (FIS; Fisk et al., 1994). The fatigue symptom inventory (FSI) and multidimensional fatigue symptom inventory (MFSI; Stein et al., 1998) both contain an item regarding sexual activity, and we suggest that a *sexual* component of fatigue could be conceptualized as further dimension. While sexual dysfunction, especially hypoactive sexual desire, is often experienced in chronic fatigue syndrome (CFS), the current understanding of this phenomenon is limited (Blazquez et al., 2009). An improved assessment of fatigue that includes the sexual domain might provide useful in addressing this problem. Another distinction that can be made is between *state* fatigue (referring to the momentary sensation of fatigue), *trait* fatigue (the overall disposition of sensation of fatigue), *prolonged state* fatigue (general tendency of sensation of fatigue within a defined time period), and *pathological fatigue* (when the general tendency of sensation of fatigue is perceived to interfere with everyday life; for a full description see Skau et al., 2021).

Penner and Paul (2017) also suggest that differentiating between primary, secondary, and comorbid fatigue is of clinical importance: symptoms of primary fatigue can be considered part of the underlying disease and emerge independently from other comorbidities, as it is the case in multiple sclerosis (MS). On the contrary, secondary fatigue results from other concomitant circumstances or disorders: potential contributing factors to fatigue are sleep disorders, reduced physical activity, pharmacotherapy, anaemia, thyroid dysfunction, and psychiatric disorders, most notably depression, whose co-occurrence with fatigue is often difficult to disentangle (Penner and Paul, 2017). Lastly, fatigue can be comorbid with a primary neurological disease, but not being causally related to it or to the presence of concomitant diseases or circumstances. In this case it is defined as comorbid fatigue. However, these concepts are theory-driven and based on causal assumptions that cannot be definitely verified. Because of the desiderata *neutrality*, whereby a definition should not depend on any particular theory, and *phenomenon focus*, according to which a definition should involve explanations to a minimal extent (Skau et al., 2021), we believe that a descriptive approach with respect to comorbidities is more helpful, especially if considering our current etiological understanding. For example, MS with fatigue rather than primary fatigue or sleep disorder with fatigue rather than secondary fatigue. The discovery of a biological mechanism underlying these three dimensions of fatigue would cause these definitions to lose their utility.

Notably, previous literature has also attempted to classify fatigue based on its origins, either central or peripheral (DeLuca, 2005). Peripheral fatigue is defined by Chaudhuri and Behan (2000, p. 34), as “the inability to sustain a specified force output or work rate during exercise,” while central fatigue is defined as “the failure to initiate and/or sustain attentional task (‘mental fatigue’) and physical activities (‘physical fatigue’) requiring self-motivation (as opposed to external stimulation)” (Chaudhuri and Behan, 2000, p. 35). Peripheral fatigue is therefore based on a reduction in objective performance originating from peripheral factors such as failure of neuromuscular transmission, metabolic defects of the muscles, or a peripheral circulatory failure (Chaudhuri and Behan, 2000). Central fatigue on the other hand, results from changes in the central nervous system (CNS), and it is characterized by different dimensions, as previously described (Chaudhuri and Behan, 2000). Those dimensions can then refer to both an objective and a subjective aspect (e.g., objective central mental fatigue would be a reduced performance in cognitive tasks, while subjective central mental fatigue would be the subjective feeling of having difficulties concentrating; Karshikoff et al., 2017). While it is important to distinguish between a subjective and objective part of fatigue, since those two components are often not identical or even closely related (Penner and Paul, 2017), defining a concept on the base of its presumptive origin is problematic. This again relates to the *neutrality* and to the *phenomenon-focus* desiderata (Skau et al., 2021). In this case, the discovery of a biological mechanism underlying both central and peripheral fatigue would cause these definitions to lose their utility. Therefore, as with primary, secondary, and comorbid fatigue, we believe a descriptive approach would be more helpful. Rather than, for instance, peripheral fatigue, which implies causality, fatigue comorbid with peripheral disorder would be preferable.

**Figure 1:**
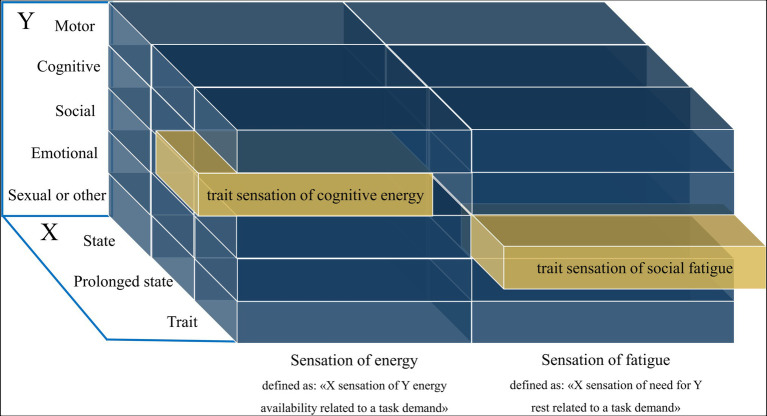
Visualization of the definition of fatigue and energy sensations. The X-axis contains the state (in this moment), prolonged state (general tendency within a defined time period), and trait (an overall disposition) dimensions. The Y-axis contains the possible domains of sensations of fatigue or energy.

Basing on these previous definitions and theoretical considerations, we propose the following definition for *sensation of fatigue*: “X sensation of need for Y rest that is related to a task demand” (see Figure 1). X constitutes the reference time period (*state*, *prolonged state*, and *trait*), while Y refers to the domain in question such as *motor*, *cognitive*, *social*, *emotional*, *motivational*, and *sexual*. For example, in the cognitive domain of fatigue that refers to the sensation at this moment, the definition would be as follows: “State sensation of need for cognitive rest that is related to a task demand.” Unlike the definition of Skau et al. (2021), the idea of a mismatch between effort expended and actual performance is not present in our definition. This is because we find its component (actual performance) does not allow to properly separate the sensations of fatigue from an objective criterion, and belong rather to the objective part of fatigue.

The sensation of fatigue has often been conceptualized as the opposite of *sensation of energy*, i.e., a sensation of a “lack of energy” suggesting that they are two ends of a single bipolar sensation (Fatigue: United States national library of medicine; 2018). However, recent work indicated that they are better conceptualized as two separate but related constructs, with a different underlying neurobiology and therefore different predictors for each of the two states (Loy et al., 2018; Boolani et al., 2019). *Energy*—not *sensation of energy*—has been defined by Loy et al. (2018) as an individual’s potential to perform mental and physical activity, with synonyms that include “vigour,” “vitality,” “lively,” and “full of pep.” A sensation of energy would hence be a sensation of one’s potential to perform mental and physical activity. We think that the term “potential” is not precise enough to avoid multiple interpretations and propose the term “energy availability.” Finally, we think that this definition does not fulfil the previously mentioned broadness desideratum, since it contains only the mental and physical dimension, while other aspects such as *social*, *emotional*, *motivational* and *sexual* energy are not included. In addition, the reference time period such as *state*, *prolonged state* and *trait* are missing. Therefore, based on the definition of energy from Loy et al. (2018), we define *sensation of energy* as: “X sensation of Y energy availability related to a task demand.” Here, X can constitute the reference time period (*state*, *prolonged state* and *trait*) and Y a domain of interest such as *motor*, *cognitive*, *social*, *emotional*, *motivational* and *sexual* (see Figure 1). It is important to note that at the core of this definition there is energy *availability*, not the energy that is consumed. For example, a state of high arousal (e.g., due to a stressor) would be characterized by a mobilization of energy resources and an increased energy consumption, but not necessarily by a sensation of energy availability.

### Defining Sensations of Fatigability and Energizability

As mentioned is the previous section, fatigue is not only characterized by a subjective sensation, but also by an objective performance-based dimension. The latter is commonly referred to as *fatigability* (Skau et al., 2021). Fatigability is related to a decrease in a performance criterion over time, which again can be in different domains, such as cognitive or physical fatigability (Skau et al., 2021).

**Figure 2:**
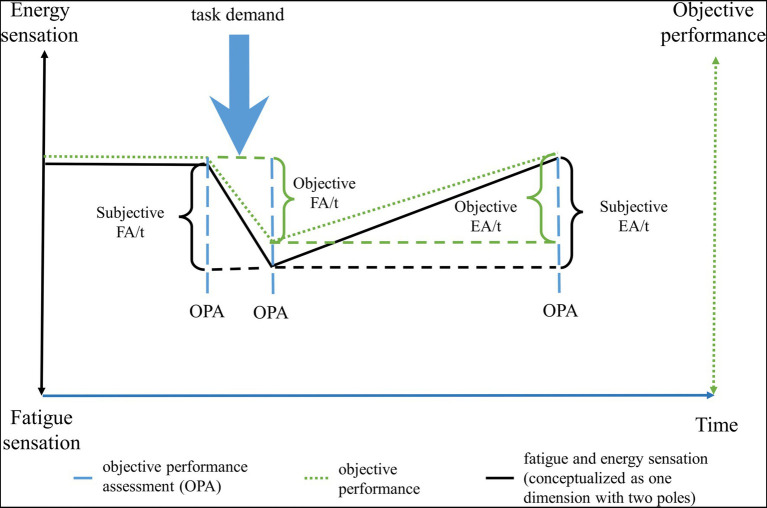
The change over time of fatigue and energy sensations, and of objective energy levels following a cognitive/physical/emotional demand. The black solid line corresponds to the subjective changes over time, while the green dotted line to the objective changes over time. To calculate the changes in objective performance, an assessment is done at the time points where the dashed blue lines are. Note that the subjective changes may not always reflect the objective changes in energy levels, as is evidenced by the slightly courses of the black and green lines. FA, fatigability; EA, energizability; OPA, objective performance assessment; and t, time.

The concept of the *need for recovery* has been introduced as well, initially consisting of the need to recuperate from work-induced fatigue (Jansen et al., 2002). This concept involves the intensity of work-induced fatigue, but also the time period required to return back to a normal level of functioning (Jansen et al., 2002). Further studies should examine this concept of the perceived need for recovery, including measures of not only the time necessary to recuperate, but also the time at which a sensation of fatigue arises. This may better clarify the temporal components of fatigue, hence having not just state or trait, but also the speed through which a sensation of fatigue arise and dissipate. We propose that these temporal components, such as the speed through which someone becomes fatigued or recuperates after an effort constitute their own dimensions, separate from the sensation of fatigue. Here, to define these time-dependent sensations, we introduce two new concepts: a subjective component of fatigability, and the concept of energizability (see Figure 2). As mentioned, *objective fatigability* is related to a decrease in a performance criterion over time (Skau et al., 2021). Similarly, we propose that the increase in sensation of fatigue per time unit while engaging in a defined task demand is a measure of *subjective fatigability*. Next, we propose *energizability* as a measure of individual differences concerning the time necessary to return to a baseline level of functioning. This dimension can be differentiated in subjective and objective energizability. *Objective energizability* is the time necessary to return to the baseline of a measurable performance criteria, while *subjective energizability* is the time necessary to reach a baseline sensation of energy (see Figure 2 for an illustration of these new concepts).

## Energy and Fatigue Sensation: Two Separate Constructs or Two Ends of One Dimension?

In disease-related research numerous different fatigue questionnaires are used, most of which assess fatigue with a multidimensional approach, but few include both energy and fatigue as distinct subscale (Hjollund et al., 2007). Among the most common fatigue measures, only the profile of mood states (POMS; Heuchert and McNair, 2012) has enough indicators of energy and fatigue for factor analysis, with eight vigour items in the 65-item original form and five in the 30-item short form, and seven fatigue items in the original form and five in the short form (Loy et al., 2018). Supporting the idea that sensations of fatigue and energy (vigour, in this case) are different constructs, exploratory and confirmatory factor analyses have found the vigour and fatigue subscales to be independent (Boyle, 1987; Gibson, 1997; Heuchert and McNair, 2012). Furthermore, when looking at the commonly used measure Profile of Mood States-Adolescents (POMS-A; Terry et al., 1999), there is a positive significant correlation between the PANAS positive affects scale (Watson et al., 1988) and the vigour subscale. The PANAS is a self-report measure of mood that contains two 10-items scales, the Positive and Negative Affect scale (Watson et al., 1988). However, there is no significant correlation between positive affect and fatigue, while the PANAS negative affects scale significantly correlates with the fatigue subscale, but not with the vigour subscale (Terry et al., 2003). The MFSI (Stein et al., 1998) and its short form, the MFSI-SF (Stein et al., 2004), has a vigour subscale as well. However, this subscale includes not only items measuring sensations of energy (e.g., “I feel energetic”), but also items such as “I feel calm,” and “I feel relaxed” (Stein et al., 1998). These findings indicate that, when assessing both sensations of energy and fatigue with a sufficient number of appropriate items, they appear to be best described as two separate constructs. In addition, sensations of energy were found to be related with positive affect, while sensations of fatigue with negative affect.

### Predictors of Sensation of Energy and Fatigue Are Overlapping but Different

In a cross-sectional study of 77 graduate health sciences students (Boolani et al., 2019), sensation of energy (termed also *feelings of energy*) were found to be predicted by higher sleep quality, higher muscle oxygen saturation, lower resting metabolic rate, and cognitive function. Sensation of fatigue (termed also *feelings of fatigue*) were predicted by sleep quality as well, but the other predictors differed: these consisted of more time spent sitting and perceived mental workload on non-school days. Predictors of fatigue accounted for 46.1% of the variance in fatigue, while energy predictors accounted for 22.7% of the variance in feelings of energy (Boolani et al., 2019). The authors suggest that these results support the view that these two sensations, while having overlapping aspects, are better conceptualized as separate constructs. However, more research is needed to confirm this, possibly including longitudinal measures, and further biomarkers of energy metabolism such as mitochondrial function, as also proposed by Boolani et al. (2019).

It has been proposed that sensation of energy evolved as part of an approach-oriented system (e.g., hunting, finding a sexual partner), with dopamine and norepinephrine playing a role in its regulation (Loy et al., 2018). On the other hand, sensations of fatigue might have been part of an avoidance-oriented system that served the purpose of promoting rest and recovery from injuries or illnesses. This system is proposed to be linked with serotonin, histamine, and the levels of inflammatory cytokines (Loy et al., 2018). For instance, the reuptake inhibitor D-amphetamine was found to increase feelings of energy, but did not have a significant effect on feelings of fatigue (measured using the POMS questionnaire; Lott et al., 2005). Methylphenidate, in the context of treating cancer fatigue, showed inconsistent results, as evidenced by the meta-analysis of Gong et al. (2014): it was found to improve scores of the Functional Assessment of Cancer Therapy-Fatigue (Cella et al., 1993), but did not significantly improve fatigue as measured by the Brief Fatigue Inventory (Mendoza et al., 1999). This could at least in part be explained, as Loy et al. (2018) suggest, by the fact that this dopaminergic drug would lead to increases in sensation of energy rather than decreases in sensations of fatigue. This was shown by the improvement on the Functional Assessment of Cancer Therapy-Fatigue scores, which contains items measuring energy sensation. No difference was found on the Brief Fatigue Inventory which may be due to the items that only assess sensation of fatigue.

Supporting this view, Loy et al. (2018) also presents studies (i) on the effects of caffeine, whose administration increased sensations of energy but not of fatigue (Amendola et al., 1998), or increased sensations of energy more than those of fatigue (Maridakis et al., 2009a,b), (ii) on genetic polymorphisms in the dopamine receptor gene DRD4, which is related to a decrease in energy with mental work but not fatigue (Lim et al., 2012), and (iii) on catecholamines depletion using *α*-methyl-*para*-tyrosine (AMPT; Verhoeff et al., 2003). Here, sensations of energy measured through the continuous visual analog scale (VAS; Bond and Lader, 1974) and POMS subscale Vigour decreased following AMPT administration, but sensations of fatigue (assessed on the POMS subscale Fatigue), increased as well, albeit to a lesser degree (Verhoeff et al., 2003). This may indicate that, as mentioned previously, sensations of fatigue and energy are not entirely separate, but that dopamine and other catecholamines also play a role for sensation of fatigue. In fact, a dopamine imbalance has been proposed to be responsible for the emergence of fatigue in MS and other neurological disorders (Dobryakova et al., 2015). Evidence from behavioural pharmacology in support of this hypothesis is mixed: methylphenidate, a dopamine agonist, was found to reduce fatigue in small studies with Parkinson’s and CFS patients (Blockmans et al., 2006; Mendonça et al., 2007), but not for MS patients (Cameron and McMillan, 2017). On the other hand, amantadine, another drug thought to improve fatigue through a dopaminergic mechanism (Brenner and Piehl, 2016; Tur, 2016), was found to have substantial effects on fatigue in MS (see Penner and Paul, 2017 for a review), even when measuring it using an instrument without energy items, like the Fatigue Severity Scale (FSS; Krupp, 1989).

Serotonin is thought to be related especially to sensation of fatigue (Loy et al., 2018). In support of this idea neuroimaging studies on MS (Hesse et al., 2014) and CFS patients (Yamamoto et al., 2004; Cleare et al., 2005) suggest that altered serotonergic neurotransmission might contribute to fatigue. A PET-imaging study by Pavese et al. (2010) on patients with Parkinson’s disease found significant differences in serotonin transmission between individuals with and without fatigue, but no differences in dopamine function. In line with these findings (i) variations in serotonin genes—assumed to result in enhanced activity of the serotonergic system—were found in CFS patients (Smith et al., 2008). (ii) Decreased blood serotonin in women in the premenstrual phase was linked to enhanced negative mood and to sensation of fatigue (Kikuchi et al., 2010). (iii) Variations of serotonin levels were observed following changes in sun luminosity which might be the cause of seasonal changes in fatigue (Harris and Dawson-Hughes, 1993; Lambert et al., 2002).

Another neurotransmitter, which has been suggested to influence fatigue, is histamine (Loy et al., 2018); with two small studies showing that altering histamine levels had an impact on the sensation of fatigue (Sasahara et al., 2015; Loy and O’Connor, 2016). Vitamin D is also thought to play a role for fatigue: low levels of vitamin D have been found to be associated with fatigue (Knutsen et al., 2010), and its normalization has been shown to improve fatigue symptoms’ severity (Roy et al., 2014). In line with the assumed role of serotonin in fatigue, vitamin D contributed to maintain an optimal brain serotonin concentration (Sabir et al., 2018; Wang et al., 2020). It is widely accepted, that the complex phenomenon of fatigue cannot be traced back to a single neurotransmitter, but rather to the interaction of multiple neurotransmitter systems (Meeusen et al., 2006; Dobryakova et al., 2015). For example, alterations of the endogenous opioidergic system as well as melatonin are also thought to play a role for CFS pathophysiology and hence might play a role in fatigue (Anderson and Maes, 2020).

### The Acquiescence Effect May Contribute to Differential Links With Predictors

Taken together, behavioural and biological predictors of sensations of energy and fatigue seem to differ indicating to two separate constructs. However, an important methodological issue to address in this debate concerns the acquiescence effect (Messick, 1966). This refers to differences of participants in the general tendency to provide affirmative answers to the items of a questionnaire, regardless of the content of the questions (Hinz et al., 2007). When a questionnaire contains items assessing both ends of a bipolar construct, such as positive and negative affectivity, the acquiescence effect can lead to the dimensions not being negatively correlated, but instead appearing as statistically independent from one another (Hinz et al., 2007). This effect has already been observed in mood research, where the aforementioned positive and negative affectivity were found, contrary to expectations, to be independent (Watson and Clark, 1997; Russell and Carroll, 1999). There is also evidence for this effect in the MFI (Hinz et al., 2007). The acquiescence effect can be avoided when items are presented in a bipolar way with verbal explanations at both ends of the scale, instead of presenting unipolar items. Unfortunately, the latter is the case in most fatigue questionnaires (e.g., MFSI, POMS), and might provide a confounding factor when trying to determine whether fatigue and energy are in fact two separate constructs.

## Energy Metabolism and Its Regulators

In this section we shortly illustrate mitochondrial function, which is at the basis of energy production for the organism, and its assessment methods. We then present important regulators of the energy metabolism, such as hormones, the hypothalamus, the autonomic nervous system, or brain derived neurotrophic factor.

### Mitochondria and Their Assessment: A Brief Overview

Energy metabolism, and mitochondrial function in particular, are at the basis of the structure and function of the human body, powering growth, healing, and the processes necessary to adapt to the changing external environment, such as stress adaptation, or allostasis (Picard et al., 2018).

In living organisms, energy is present as heat, and as chemical energy such as adenosine triphosphate (ATP; Picard et al., 2018). Briefly illustrated, energy production in the body can happen in the mitochondria in two different ways, aerobic and anaerobic respiration. Glucose, the main energy source for cellular metabolism, is catabolized in three subsequent processes to produce ATP. The first process is glycolysis, where glucose is converted into pyruvate, and a low amount of ATP is produced (Bonora et al., 2012). Then, pyruvate is converted to acetyl coenzyme A, which enters the tricarboxylic acid cycle (TCA) or Krebs cycle. This results in the production of NADH and FADH_2_, which are used during the last process, oxidative phosphorylation (Bonora et al., 2012). Here, electrons carried by NADH and FADH_2_ are passed across the electron transport chain (ETC), where O_2_ serves as the final electron acceptor, to pump protons out of the inner mitochondrial membrane (Mavioglu et al., 2021). The resulting proton gradient is necessary to synthesize large amounts of ATP by mitochondrial ATP synthase (Bonora et al., 2012). An adequate supply of O_2_ is therefore crucial for oxidative phosphorylation (Mavioglu et al., 2021). In anaerobic respiration, glucose is converted to pyruvate during glycolysis. Pyruvate is reduced to lactate during a process called lactic fermentation, and the final quantity of ATP produced is lower than that produced during aerobic respiration (Bonora et al., 2012).

Mitochondria are not only responsible for ATP production, but also for cellular thermogenesis (Picard et al., 2018). In fact, some patients with mitochondrial disease appear to have problems with regulating body temperature, and to exhibit persistently lower brain temperature (Rango et al., 2014). Part of the heat generated by mitochondria is released during the process of converting foods to utilizable energy, and from energy mechanical work (Galgani and Ravussin, 2011). Through the process of mitochondrial uncoupling, chemical reactions in the mitochondrial matrix become uncoupled from ATP synthesis and release heat, thus increasing core body temperature (Chouchani et al., 2016). This process occurs mostly in brown adipose tissue (BAT) and was known to be the main process contributing to thermoregulation in infants and small mammals (Cannon and Nedergaard, 2004). Brown fat was more recently discovered in adult humans as well, even though it decreases with age, spurring new interest in the possibility of its central role for obesity and diabetes (Nedergaard and Cannon, 2010; Halpern et al., 2014).

Mitochondrial function can be characterized by *in vitro* and *in vivo* approaches. State of the art approaches analyse (1) maximal ATP synthesis, (2) maximal oxygen consumption, (3) mitochondrial coupling, and (4) protein synthesis rates (Lanza and Nair, 2010). High-resolution respirometry is an emerging *in vitro* methodology, which allows to measure the amount of oxygen consumed for a given amount of ATP synthesized by the mitochondria, thus providing an assessment of the efficiency of oxidative phosphorylation (Lanza and Nair, 2010). Near infrared and magnetic resonance spectroscopy (NIRS, MRS), which are becoming more accessible and allow for non-invasive *in vivo* measures, can provide an assessment of both maximal mitochondrial oxygen consumption and maximal ATP synthesis capacity (Lanza and Nair, 2010).

The basal metabolic rate (BMR) consists of the total energy necessary to maintain the bodily processes necessary for life at homoeothermic temperature at rest and with the digestive system inactive (Galgani and Ravussin, 2011). Examples of such processes include maintaining gradient concentrations between cellular compartments, muscle tone, protein synthesis and degradation, and RNA and DNA turnover (Galgani and Ravussin, 2011). While measuring the BMR requires very specific conditions, the resting metabolic rate (RMR) is more easily accessible (Galgani and Ravussin, 2011). The BMR can be accelerated by physical activity or stimulation of metabolic processes, therefore resulting in an increased heat production (McAninch and Bianco, 2014).

### Regulators of Energy Metabolism

Energy metabolism is regulated, among other factors, by the autonomic nervous system (ANS), thyroid hormones (TH), and the hypothalamus (Kalsbeek et al., 2010; McAninch and Bianco, 2014). For instance, following environmental stimuli such as food or temperature, or hormonal stimuli such as leptin, the hypothalamus modulates the sympathetic output and secretion of TH, which in turn can regulate energy metabolism by affecting different organ systems (McAninch and Bianco, 2014). TH and sympathetic activity modulate blood pressure and heart rate, glucose homeostasis, endogenous glucose production in the liver, and thermogenesis in the aforementioned BAT (McAninch and Bianco, 2014). The thyroid hormone T3 can even induce mitochondrial biogenesis (Lee et al., 2012). An example of hypothalamic control of energy metabolism is the circadian regulation of glucose metabolism: the daily rhythms of plasma glucose concentrations, glucose uptake, and insulin sensitivity are regulated by the hypothalamic biological clock (suprachiasmatic nuclei), which has projections in both the neuroendocrine and pre-autonomic hypothalamic neurons (Kalsbeek et al., 2010). The importance of the ANS for the energy metabolism can also be evidenced by the fact that its dysregulation can predict the metabolic abnormalities seen in the metabolic syndrome, such as increases in blood pressure and decreases in HDL cholesterol (Licht et al., 2013). Analogously, abnormalities in TH levels strongly affect metabolism: severe hypothyroidism can reduce the total body energy expenditure by as much as 50% (Oppenheimer et al., 1987).

Not just TH regulate energy metabolism, other hormones include insulin, glucagon, somatostatin, catecholamines such as epinephrine, glucocorticoids such as cortisol, ACTH, growth hormone, sex steroids such as estrogens (Barth et al., 2007; López and Tena-Sempere, 2015; Qaid and Abdelrahman, 2016; Picard et al., 2018), and the aforementioned leptin. Notably, the synthesis of all steroid hormones takes place in the mitochondria, and, reciprocally, glucocorticoids and other steroid hormones can influence mitochondrial function (Picard et al., 2018). Another important regulator of energy metabolism is brain-derived neurotrophic factor (BDNF). In the hypothalamus, BDNF can inhibit food intake and regulate peripheral energy metabolism (Rothman et al., 2012). Furthermore, BDNF play an essential role for the development of the ANS, especially for the formation of synaptic connectivity with the periphery, but also for the ANS control of cardiovascular functions, regulating parasympathetic and/or sympathetic inputs to the heart (Rothman et al., 2012). Finally, BDNFs mediate the positive effects of exercise and dietary energy restriction on cognitive functions, synaptic plasticity, neurogenesis, antioxidant production, mitochondrial biogenesis, enhanced cellular energy metabolism, and DNA repair (Rothman et al., 2012). While the hypothalamus and the brainstem have a central role in the control of energy metabolism (Roh and Kim, 2016), the amygdala has been proposed to be a complementary circuit for the control of energy balance, especially in an interplay with sex hormones (Pineda et al., 2021).

## The Link Between Fatigue and Energy Metabolism

In the following, we will review the link between fatigue—as the most investigated energy-related sensation—and different components of energy metabolism. These components include (i) mitochondrial bioenergetics, (ii) oxygen, that are both directly involved in ATP synthesis, (iii) the ANS as a main regulator of energy metabolism, and (iv) inflammation which is bi-directionally related with mitochondrial bioenergetics.

### Role of Mitochondrial Bioenergetics for Fatigue

In this section we review evidence concerning fatigue as a correlate of mitochondrial bioenergetics. This link can be seen in the case of mitochondrial diseases, where fatigue is a core symptom (Filler et al., 2014; Parikh et al., 2019). Fatigue is also strongly related to mitochondrial bioenergetics (Karabatsiakis et al., 2014; Brand et al., 2020). In studies on primary genetic mitochondrial diseases, fatigue was reported by up to 100% of patients (Parikh et al., 2019). Mitochondrial dysfunction can be of primary origin, resulting from inherited mitochondrial DNA (mtDNA) mutations, or from mutation of nuclear (nDNA) genomes, since nDNA encodes respiratory chain subunits and all the proteins responsible for mtDNA maintenance, transcription, and copy number control of the mitochondrial genome (Cohen and Gold, 2001; Rusecka et al., 2018). Mitochondrial dysfunctions can also be of secondary origin, as the results of external factors such as environmental and pharmacological toxins damaging the mtDNA (Cohen and Gold, 2001). For CFS, a study showed that all patients examined suffered from measurable mitochondrial dysfunctions (Booth et al., 2012). Several markers of mitochondrial function that might have an association with fatigue have been identified, including mitochondria structure, mitochondrial function, mitochondrial energy metabolism, and immune response (see Filler et al., 2014 for a review).

#### Mitochondrial Structure

The earliest evidence of structural changes in mitochondria structure comes from a study on CFS patients by Behan et al. (1991), where branching and fusion of cristae within skeletal muscle cells’ mitochondria were observed, plus variable shape and size. Branching of cristae and mitochondrial hypertrophy were found in a subsequent study by Behan et al. (1995). Condensation of cristae were also found by Lawson et al. (2016), but no differences in shape and size of mitochondria in peripheral blood mononuclear cells. The conclusion from Lawson et al. (2016) and Behan et al. (1991), was that increased energy demands in CFS caused this branching of cristae. Other studies investigating the relationship between fatigue and mitochondrial number, shape and/or size found no significant differences in shape and size of mitochondria in CFS patients, in contrast to controls (Edwards et al., 1993; Plioplys and Plioplys, 1995a; Behan et al., 1999).

#### Mitochondrial Function

Studies on mitochondrial functions have covered different aspects. The first is mitochondrial enzymes, with Coenzyme Q10 (CoQ10) being one of the most studied. It is an important antioxidant in mitochondria and has been shown to restore respiration (James et al., 2005; Wood et al., 2021). Genetic disorders that result in lowered levels of CoQ10 lead to severe debilitating symptoms and some forms of mitochondrial disease (Emmanuele et al., 2012; Rodríguez-Aguilera et al., 2017; Stefely and Pagliarini, 2017). Different studies have demonstrated that plasma levels of CoQ10 are lower in CFS patients in comparison to healthy controls (Kurup and Kurup, 2003a,b; Maes et al., 2009a,b; Castro-Marrero et al., 2013), as well as in depressed patients with CFS (Maes et al., 2009a,b). Finally, CoQ10 plasma levels show an inverse relationship with CFS severity scores (Maes et al., 2009a). Studies on CoQ10 supplementation for fatigue provide contrasting results (Mehrabani et al., 2019; Wood et al., 2021). This might be explained by the low bioavailability of CoQ10 due to its low water solubility and high molecular mass, plus the fact that the extent to which CoQ10 enters cells is still unknown (Wood et al., 2021).

Studies comparing other mitochondrial enzymes in CFS patients against healthy controls have showed conflicting results: two studies found a significant reduction in citrate synthase, a crucial enzyme in the citric acid cycle, in muscle biopsy samples (McArdle et al., 1996; Smits et al., 2011). McArdle et al. (1996) found a reduction in succinate reductase and cytochrome-c oxidase, two of the four mitochondrial transmembrane enzyme complexes of the electron transport chain (Tymoczko et al., 2011), while another study did not find a significant difference in cytochrome-c oxidase and myoadenylate deaminase (MAD; Edwards et al., 1993).

Oxidative stress consists of the imbalance between the production of reactive oxygen species (ROS) and antioxidant defences, resulting in increased amounts of free radicals (Betteridge, 2000; Wood et al., 2021). Oxidative stress is involved in the aging process and several pathological conditions including cardiovascular diseases, neurodegenerative diseases, and cancer (Liguori et al., 2018). Mitochondria are a major producer of ROS and are in turn very susceptible to damage from oxidative stress. This said, they possess also a sophisticated defence system against ROS consisting of many enzymes and coenzymes such as vitamin E, the aforementioned CoQ10, and cytochrome-c plus cytochrome-c oxidase (Starkov, 2008; Wood et al., 2021). Damages and dysfunctions in this removal system, such as low levels of a ROS-removing enzyme, cause ROS levels to increase (Starkov, 2008; Wood et al., 2021). High oxidative stress has been found in CFS patients in comparison to healthy controls (Kurup and Kurup, 2003a,b). CFS patients showed increased levels of ROS, decreased levels of antioxidants in the plasma, serum and in mitochondria (Kurup and Kurup, 2003a,b; Castro-Marrero et al., 2013), increased markers of oxidative damage, and increased indicator of oxidative stress including decreased hypoxanthine in blood and increased allantoin in urine (Armstrong et al., 2015). Other studies found increased markers of oxidative stress at baseline and after physical exercise in CFS patients with a history of high-level sport practice and/or severe infection (Jammes et al., 2012). In addition, CFS patients who relapsed in comparison to those who were in remission showed increased levels of oxidative stress (measured through serum vitamin E concentration; Miwa and Fujita, 2010). Markers of oxidative stress were also associated with symptom severity of CFS (Richards et al., 2000). Lastly, when comparing fatigued systemic lupus erythematosus (SLE) patients with non-fatigued SLE patients, Segal et al. (2012) found higher levels of plasma F_2_-isoprostane, an *in vivo* measure of oxidative stress.

#### Mitochondrial Energy Metabolism

Dysfunctions in mitochondrial energy metabolism were found in CFS patients when using the ATP profile test, indicating that ATP production was impaired (Myhill et al., 2009; Booth et al., 2012). In addition, with increasing mitochondrial function, symptoms of fatigue decreased (Myhill et al., 2009). A study investigating mitochondrial respiration in the peripheral blood mononuclear cells of major depression patients against controls showed that mitochondrial respiration is significantly reduced in acutely depressed individuals, with a significantly lower routine respiration, maximal uncoupled respiration, spare respiratory capacity, and ATP-turnover-related respiration (Karabatsiakis et al., 2014). Mitochondrial respiration was shown to negatively correlate with depressive symptom severity, including loss of energy and fatigue, measured through the respective BDI-II subscales (Karabatsiakis et al., 2014). Similarly, Booth et al. (2012), concluded in a study with 138 CFS patients using the ATP profile test, that the major immediate causes of mitochondrial dysfunction are: (i) lack of essential substrates and (ii) partial blocking of the translocator protein sites and/or oxidative phosphorylation and the reactions leading up to it.

Tomas et al. (2017) found that CFS patients had lower measures of oxidative phosphorylation in peripheral blood mononuclear cells in comparison to controls, including lower reserve capacity and lower maximal respiration. As the authors suggest, this indicates that CFS patients are not capable of the same levels of respiration as healthy subjects in a situation of cellular stress, so in the event of increased energy demands such as physical exercise or acute stress, CFS patients are unable to increase their respiration rate and fulfil those demands (Tomas et al., 2017). Missailidis et al. (2020) also suggested that mitochondria are unable to increase energy from baseline to meet heightened energy demands.

Despite these results showing CFS patients suffering from impaired energy production, three studies examining aerobic respiration and respiratory chain complex activity found no differences between CFS patients and controls (Behan et al., 1999; Vermeulen et al., 2010; Smits et al., 2011). Lawson et al. (2016) and Tomas et al. (2019), again in the context of CFS against healthy controls, found no differences in complexes of the electron transport chain. They therefore suggested that dysfunctions might then be due to external stimuli, a pathological mechanism by which more ATP is produced by non-mitochondrial sources such as glycolysis (Lawson et al., 2016), or other causes upstream of the mitochondrial respiratory chain (Tomas et al., 2019), for example, oxidative damage (Morris and Maes, 2014).

Another aspect of mitochondrial energy metabolism, fatty acid metabolism, has been investigated by examining carnitine levels in CFS and MS patients (Filler et al., 2014). Different studies showed that dysfunctional carnitine levels were present in these conditions, but the specific dysfunctions were heterogeneous: lower acylcarnitine serum levels were present in CFS patients compared to healthy controls in two studies (Kuratsune et al., 1994; Plioplys and Plioplys, 1995b), as well as lower serum levels of total carnitine (Plioplys and Plioplys, 1995b). Meanwhile, other studies found no differences in these indicators for CFS (Soetekouw, 2000; Reuter and Evans, 2011) and MS patients (Fukazawa et al., 1996) in comparison to healthy controls. While not finding differences in the l-carnitine, total carnitine, or total acylcarnitine plasma levels of CFS patients and controls, Reuter and Evans (2011) found six acylcarnitine subtypes were significantly lower and two other subtypes were significantly higher in the plasma levels of CFS patients. Regarding the relation with fatigue symptoms, higher acylcarnitine levels are negatively correlated with CFS symptoms (Kuratsune et al., 1994; Plioplys and Plioplys, 1995b), while this relationship was not found for free l-carnitine serum levels in the study of Kuratsune et al. (1994). Another study found that higher free and total carnitine serum levels were associated with lower fatigue severity (Plioplys and Plioplys, 1995b). Lastly, a meta-analysis study on carnitine supplementation as a treatment for cancer related fatigue did not found it to be effective in reducing fatigue (Marx et al., 2017). Notably, only three studies were included in the quantitative meta-analysis, many others were non-randomized, open-label or presented other risks of bias (Marx et al., 2017).

#### Autoimmune Response to Mitochondria

A dysfunctional, autoimmune response to mitochondria has been hypothesized to cause chronic fatigue (Hokama et al., 2008, 2009). Prior or ongoing infections such as the Epstein–Barr virus (EBV) have previously been associated with CFS pathoetiology and/or pathophysiology, with the viruses driving and/or interacting with dysregulated immune responses and significantly modulating mitochondrial function (Anderson and Maes, 2020). In fact, hijacking the host mitochondria is a fundamental aspect that allows the survival and replication of most viruses (Anderson and Maes, 2020).

The damage or stimulation of the mitochondria through viral infection is thought to result in the release of phospholipids, which would then induce the production of an autoimmune antibody (antiphospholipids; Hokama et al., 2008). Corroborating this hypothesis, it has been shown that the serum of CFS patients contained such antibodies (Hokama et al., 2008, 2009). Another study found that a significant proportion of CFS patients were producing EBV antibodies, and their levels negatively correlated with CFS symptoms (Williams et al., 2019). Preliminary evidence from Vernon et al. (2006) showed that people who developed post-infective fatigue after EBV infection presented gene expression profiles different than those of people who recovered without complications. Several of the differentially expressed genes affected different aspects of mitochondrial function (Vernon et al., 2006).

### Role of Oxygen for Fatigue

As described in the Energy and Fatigue Sensation: Two Separate Constructs or Two Ends of One Dimension? section, oxygen plays a crucial role for oxidative phosphorylation. Reduced oxygen delivery should lead to a decreased oxidative metabolism and fatigue. Blunted O_2_ delivery exacerbates muscular fatigue, while conditions of hyperoxia attenuate the rate at which fatigue develops (Amann and Calbet, 2008). Insufficient oxygen delivery, resulting from modifications in arterial O_2_ content, blood flow, or both, is thought to affect both fatigue at the level of the CNS and of the working muscles (Amann and Calbet, 2008). In the case of CFS, reduced oxygen delivery to muscles and reduced oxygen extraction by muscle cells in comparison to controls have been observed (Mccully and Natelson, 1999; Vermeulen and Vermeulen van Eck, 2014). A meta-analysis showed that CFS patients present a substantially reduced VO_2peak_ in comparison to apparently healthy controls (Franklin et al., 2019). The role of oxygen has been highlighted not only in the case of CFS, but also for the development of major depressive disorder (MDD; Mavioglu et al., 2021). Reduced mitochondrial energy production has been observed in MDD patients (Baxter, 1989; Videbech, 2000; Hroudová et al., 2013; Karabatsiakis et al., 2014), and alterations in mitochondrial function and biogenesis are critical factors for the pathophysiology of MDD (Klinedinst and Regenold, 2015; Bansal and Kuhad, 2016; Allen et al., 2018, 2021). Alterations in O_2_ transport caused by inflammation and oxidative stress might lead to an hypoxic response, thus explaining these changes in energy metabolism (Mavioglu et al., 2021). This impairment in the functionality of depressed patients’ cells can lead to the characteristic symptoms of this disorder, such as fatigue, lack of energy, concentration, memory, and emotion regulation problems, and dysregulated inflammatory process (Gardner, 2003; Moretti et al., 2003; Karabatsiakis et al., 2014; Firth et al., 2018). Furthermore, oxygen is a substrate not only for ATP, but also for serotonin production (Østergaard et al., 2018). Serotonin depletion is thought to play an important role for depression (van Praag, 2004) and, as previously presented, for fatigue as well (Meeusen et al., 2006; Loy et al., 2018). Both CFS and MDD may thus be conceptualized as conditions characterized by a state of reduced oxygen and ATP availability, which would lead to the development of their characteristic symptoms such as fatigue.

### Role of the ANS for Fatigue

Acute, sub-acute, and chronic fatigue are characterized by changes in autonomic function (Tanaka et al., 2015). ANS can be measured using heart-rate variability (HRV), whereby low-frequency (LF) variability is considered to indicate sympathetic nervous system activity, while high-frequency (HF) variability is vagally mediated (Akselrod et al., 1981; Tanaka et al., 2015). When acute or sub-acute fatigue are induced in healthy individuals, increased sympathetic nerve activity, and decreased parasympathetic nerve activity can be observed and correlated with subjective measures of fatigue (Tanaka et al., 2009, 2011; Mizuno et al., 2011). Sympathetic hyperactivity based on decreased parasympathetic nerve activity has been reported in CFS patients as well (see Tanaka et al., 2015 for a review). Suppressed parasympathetic activity, reflected by HRV, has been observed during deep sleep in CFS patients in comparison to controls, and was associated with poorer sleep quality and self-reported well-being (Fatt et al., 2020). Dysfunctions in autonomic cardiac regulation in CFS patients in comparison to healthy controls have been shown in the meta-analysis of Nelson et al. (2019): CFS patients were found to have higher resting heart rate, higher heart rate response to hear-up tilt test, higher orthostatic heart-rate response, higher LF/HF ratio, lower maximal heart rate, lower heart rate at submaximal exercise threshold, lower high-frequency power of HRV, and lower root-mean-square difference of successive normal R-R intervals from time-domain analysis (RMSSD; both of the latter two parameters represent primarily parasympathetic cardiac modulation). These differences, the authors conclude, suggest reduced vagal and increased sympathetic modulation of heart rate (Nelson et al., 2019). Autonomic alterations are frequent also in MS patients (de Seze et al., 2001; Kodounis et al., 2005; Merkelbach et al., 2006), and have been shown to be an independent predictor of MS-related fatigue (Krbot Skorić et al., 2019), even though a study on newly diagnosed MS patients found very mild differences in autonomic responses to stress in comparison to healthy controls, suggesting it might be connected with disease progression (Vlcek et al., 2018).

During tasks eliciting acute mental fatigue, changes in activation in the prefrontal cortex (PFC) and anterior cingulate cortex (ACC) are observed over loading time (Caseras et al., 2008; Mizuno et al., 2012; Tanaka et al., 2015; Wang et al., 2016). The PFC and ACC are also part of the network controlling the sympathetic-vagal balance (Loewy, 1990; Benarroch, 1993), with the PFC inhibiting sympathoexcitatory subcortical threat circuits (Amat et al., 2005; Thayer, 2006). Therefore, Tanaka et al. (2015) suggest that changes in these regions following acute mental fatigue may hinder the capacity of controlling the sympathoexcitatory response, which might explain the changes in autonomic function presented previously. CFS patients exhibit lower PFC activity during fatigue-inducing tasks (Caseras et al., 2008), impaired prefrontal cerebral oxygenation during physical exercise (Patrick Neary et al., 2008), and reduced bilateral grey matter volume (GMV) in the PFC (Okada et al., 2004). However, a more recent study found reduced GMV in CFS patients to be associated with increased pain rather than fatigue, not allowing a reliable association between CFS and reduced GMV (van der Schaaf et al., 2017). The PFC is thought to play an important role for fatigue in MS as well. Fatigue in MS patients has been associated with lesions in the frontal and parietotemporal white matter, grey matter atrophy in frontal regions (Sepulcre et al., 2009), reduced white matter integrity in various frontal networks (Pardini et al., 2010), alterations in functional integration between frontal cortex and subcortical structures such as basal ganglia and thalamus (Roelcke et al., 1997; Filippi et al., 2002; Derache et al., 2013), and connectivity alterations between cortical (PFC and cingulate cortex) and subcortical structures (basal ganglia, thalamus; Finke et al., 2015; Pravatà et al., 2016). It could be speculated that these alterations in the frontal regions might lead to a constant imbalance in autonomic function, with difficulties in inhibiting the sympathetic drive.

### Role of Inflammation for Fatigue

Here we will present evidence coming from clinical studies linking inflammation and the development of fatigue, followed by a section on possible mechanisms of action through which inflammation may lead to fatigue.

#### The Association Between Inflammation and Fatigue: Evidence From Physical and Mental Conditions

A third central contributor to the feelings of fatigue is thought to be inflammation (Karshikoff et al., 2017). Evidence that inflammation plays a role in the development of pathological fatigue has been found in different clinical populations such as cancer patients (Schubert et al., 2007; Bower and Lamkin, 2013). Increased concentrations of inflammatory markers such as C-reactive protein (CRP) and interleukin (IL-6) during cancer treatment were related to the development of feelings of fatigue (Bower et al., 2009; Liu et al., 2012; Xiao et al., 2016). Even after the illness, increased CRP levels have been found to correlate with overall levels of fatigue (Bower et al., 2002; Collado-Hidalgo et al., 2006; Orre et al., 2011; Zick et al., 2014). Other studies assessing fatigue multidimensionally found that inflammation in cancer patients and survivors appears to be associated with physical fatigue in particular, and less with mental fatigue (Inagaki et al., 2008; Alfano et al., 2012; de Raaf et al., 2012). In CFS, an altered immune system is thought to play a central role for the pathophysiology of this disease (Karshikoff et al., 2017). Evidence shows that, in comparison to healthy controls, production of pro-inflammatory cytokines and CRP in CFS patients is increased both at baseline and after immune stimulation (Chao et al., 1991; Patarca et al., 1994; Buchwald et al., 1997; Moss et al., 1999; Fletcher et al., 2009; Raison et al., 2009). However, not just pro-inflammatory, but also anti-inflammatory cytokines have been found to be increased in the cerebrospinal fluid in CFS patients (Peterson et al., 2015). This suggests the presence of a mixed immune response, arising from the interaction of an immune-inflammatory response system and a compensatory response system (Anderson and Maes, 2020). Variations between these two systems might explain the pathophysiological heterogeneity of CFS (Anderson and Maes, 2020). In other clinical conditions, inflammation has been associated with different facets of fatigue. For instance, in the case of type 2 diabetes, the systemic, low-grade inflammation that is present in this disorder was found to be associated with lack of motivation and mental fatigue, but not with physical fatigue (Lasselin et al., 2012). Inflammation in MS related to both physical fatigue, mental fatigue, and sleepiness (Heesen, 2006). Inflammation, measured through CRP levels, has been found to be associated with fatigue levels (Jokela et al., 2016) and to be predictive of the development of future fatigue in healthy subjects (Cho et al., 2009).

Generally, a multidimensional assessment of fatigue is lacking; if implemented it might help clarify which neuronal and physiological processes influence which aspects of fatigue (Karshikoff et al., 2017). For instance, dopaminergic transmission is sensitive to inflammation, which might explain motivational changes in fatigue conditions (Dantzer et al., 2014). Other studies have shown the depressive symptoms and adiposity to be better predictors of fatigue than inflammation markers (Lim et al., 2005; Valentine et al., 2011). However, both obesity and depression have been associated with elevated levels of inflammation (de Heredia et al., 2012; Østergaard et al., 2018), so it might be interesting to try to further disentangle these associations, possibly analysing the contribution of the previously discussed mitochondrial activity to fatigue in these conditions. Furthermore, the lack of a significant association found in some studies (e.g., Lim et al., 2005) might be explained by the fact that circulating concentrations of cytokines were measured. This represents a limitation, since circulating levels of cytokines have little functional value, and for the most part represent spillover from the site of cytokine production and action (Dantzer et al., 2014).

#### How Inflammation Might Affect Fatigue: Mechanisms of Action

When the immune system activates, cytokines are produced by the immune cells (Karshikoff et al., 2017). Cytokines not only coordinate the immune response but can also signal to the brain about immune events (Dantzer et al., 2014). They can do so *via* neuronal and humoral immune-brain communication pathways, with the former involving the activation of the vagus nerve at the periphery, which then modulates the brain targets of vagal afferents (Konsman et al., 2002). The slower humoral pathway involves the activation of brain immune cells (microglia), which in turn produce cytokines that propagate from the blood to the brain side of the blood brain barrier (Dantzer et al., 2014). Heightened levels of activity of immune-type cells of the CNS have been found in CFS, supporting the role of CNS inflammatory processes for this disorder (Nakatomi et al., 2014; Anderson and Maes, 2020). Cytokines can also inhibit the synthesis of neurotransmitters such as dopamine, norepinephrine, and serotonin (Dantzer et al., 2014), and modulate the neuroendocrine system, for instance by activating the release of cortisol, adrenocorticotropic hormone, and corticotropin-releasing hormone (Karshikoff et al., 2017). Because of the decrease in dopamine synthesis seen following inflammation, it has been suggested that this supports the idea of a dopamine imbalance hypothesis of fatigue (Dobryakova et al., 2015). This would be in contrast with the view of Loy et al. (2018), who, as previously discussed, see fatigue to be associated with serotonin. The fronto-striatal dopaminergic network is involved in reward-based decision-making, and its dysfunction following inflammation may explain the lack of motivation associated with fatigue or anhedonia in major depressive disorder (Dantzer et al., 2014). Insula activity has been shown to increase following inflammation (Harrison et al., 2009, 2015; Hannestad et al., 2012; Benson et al., 2015; Karshikoff et al., 2016). Since it is considered the central structure for the perception of interoceptive signals, it has been suggested that the subjective feelings of fatigue might be due to this increased insular activation (Karshikoff et al., 2017). Lastly, Karshikoff et al. (2017) suggest that a mental feeling of fatigue during inflammation might be due to changes in ACC functions. As partly mentioned before, increased ACC activity has been found in CFS and MS fatigued patients (Filippi et al., 2002; Schmaling et al., 2003), but also during the activation of the immune system (Capuron et al., 2005; Eisenberger et al., 2009; Harrison et al., 2009; Hannestad et al., 2012; Haroon et al., 2014). Hence, stronger ACC activity triggered by inflammation may signal a need for increased cognitive processing, thus provoking a feeling of mental fatigue (Karshikoff et al., 2017). Moreover, these inflammation-driven alterations of ACC and frontal activity might explain the autonomic imbalance found in fatigue conditions previously touched upon.

Finally, a study investigating whole-brain markers of neuroinflammation using magnetic resonance spectroscopy showed that, in comparison to healthy controls, CFS patients showed brain temperature and metabolites abnormalities across large portions of the brain, hinting to global neuroinflammation (Mueller et al., 2020). CFS patients had higher brain temperature in the right frontal cortex, right insula, right thalamus, right putamen, and cerebellum. Those temperatures were not attributable to increases in body temperature, and the right insula, right thalamus, and cerebellum were also associated with increased lactate, a by-product of anaerobic glycolysis, which is not found at high levels in the healthy brain, but it is produced by immune cells under inflammatory conditions (Mueller et al., 2020). Elevated levels of choline containing compounds (CHO), a metabolite related to neuroinflammation, were found in the bilateral ACC, left middle cingulate, right calcarine sulcus, right occipital and temporal lobes, with the most significant difference being found in the left ACC (Mueller et al., 2020). Increased levels of lactate, and therefore anaerobic energy production, were observed in numerous brain regions of CFS patients (Mueller et al., 2020). Since anaerobic ATP production is much less efficient than the healthy, aerobic mitochondrial respiration, fatigue may be explained by the cellular energy deficiency resulting from anaerobic glycolysis (Mueller et al., 2020). This picture is different from that provided by a study on patients with mitochondrial diseases, where brain temperature was lower in comparison to controls both at rest, during activation, and during recovery (Rango et al., 2014). Furthermore, the patients with the highest disruptions in mitochondrial function exhibited the lowest brain temperatures (Rango et al., 2014). This might indicate that despite both conditions being characterized by dysfunctions in mitochondrial bioenergetics, inflammation might play a more central role in the case of CFS.

## Treatments That Affect Both Energy-Related Sensations and Energy Metabolism

Physical exercise has been shown to improve energy-related sensations such as energetic arousal, calmness, burnout, emotional exhaustion, fatigue, and vigour (Puetz et al., 2008; Kanning and Schlicht, 2010; Brand et al., 2020). Exercise appears to improve sensations of energy more consistently than sensations of fatigue: a meta-analysis on the effects of a single bout of exercise found that while acute exercise homogenously increased sensations of energy, the effects for fatigue were heterogeneous, and moderated by exercise intensity and duration (Loy et al., 2013). Similarly, 6 weeks of either low or moderate chronic exercise in adults reporting persistent fatigue improved sensations of energy in both conditions. Improvements in fatigue were moderated by exercise intensity in the way that low-intensity exercise was more effective (Puetz et al., 2008). These improvements were independent of changes in aerobic fitness (Puetz et al., 2008). Exercise at moderate intensity also improved sensations of energy in people with depressive symptoms, but not sensations of fatigue (Legrand et al., 2018).

Interestingly, regular physical activity in patients with burnout improved symptoms of burnout and depression, as well as mitochondrial activity (Brand et al., 2020). Higher mitochondrial activity correlated with lower scores of burnout and depression both at baseline and in the follow-up of the study (Brand et al., 2020). Implementing an individualized activity program for CFS patients resulted in a significant reduction of different fatigue measures, an increase in mitochondrial function measured through levels of transmembrane proteins mitofusin 1 and 2 (Mfn1, Mfn2), peak VO2, and a decrease in the ratio of sympathetic to parasympathetic activity during the head-up tilt test (Kujawski et al., 2021). Interestingly, those changes in autonomic functions were related to increases in submaximal VO2 (Kujawski et al., 2021). It should be noted that such an intervention did not appear to be well-tolerated by CFS patients, since the drop-out rate was 51% (Kujawski et al., 2021). This is consistent with the idea that it might be hard for CFS patients to profit from more intensive aerobic exercise due to the severe fatigue symptoms they might experience afterwards (Puetz et al., 2008).

Improving mitochondrial bioenergetics in order to improve fatigue has been attempted through CoQ10 supplementation, but with mixed evidence (Wood et al., 2021). As previously mentioned, this might be due to the low bioavailability and the unknown extent to which CoQ10 enters the cells (Wood et al., 2021). Similarly, more evidence is needed in respect to carnitine supplementation as a treatment for MS- or cancer-related fatigue (Tejani et al., 2012; Marx et al., 2017). An audit of CFS patients who followed a tailored treatment regime that addressed their mitochondrial dysfunction (based on an ATP profile test) showed that patients who adhered to this regime exhibited significant improvements in mitochondrial function, but unfortunately no measures of fatigue were reported (Myhill et al., 2013). The treatment, in addition to a basic protocol covering sleep, nutritional supplements, a stone-age diet, and balancing work and rest, addressed either substrate or co-factor deficiency, or inhibition of mitochondrial function by chemicals (exogenous or endogenous; Myhill et al., 2013). There is also an increasing interest in the role of melatonin in mitochondrial metabolism, since exogenous and pineal melatonin have immune-dampening effects and optimize mitochondrial respiration (Anderson and Maes, 2020). Melatonin improved fatigue and activity levels in CFS patients (Heukelom et al., 2006).

In order to address mitochondrial dysfunction and inflammation in CFS, mitoprotective diets have been proposed as a treatment (Craig, 2015). These comprise of caloric restriction (CR), fasting, and ketogenic diets (Craig, 2015). Caloric restriction is often defined as a 20%–40% reduction in caloric intake (Craig, 2015), and can modulate inflammatory pathways, energy metabolism, mtDNA repair, and oxidative stress (Mattson, 2014). Fasting has also been shown to reduce subjective pain and inflammation in rheumatoid arthritis patients (Müller et al., 2001), to suppress pro-inflammatory cytokines in healthy people (Faris et al., 2012), and to reduce fatigue and weakness in cancer patients (Raffaghello et al., 2010). A ketogenic diet is a high fat, very low carbohydrate diet that can mimic the effects of fasting or CR, affecting mitochondria in a similar way as a CR diet (Craig, 2015). However, experimental trials on the effects of a ketogenic diet on fatigue are still lacking (Anderson and Maes, 2020).

Another potential therapeutic area concerns psychotropic and neurological medications that affect mitochondrial complex I and IV (Holper et al., 2019). A meta-analysis by Holper et al. (2019), which examined the effects of such medications on mitochondrial complex I and IV in the rodent brain from *in vivo* and *in vitro* studies, showed that antidepressants had heterogeneous effects, either increasing or decreasing complex I and IV, antipsychotics and stimulants decrease complex I but increase complex IV, while both complexes were preserved or enhanced by anxiolytics, mood stabilizers, antidementia and antiparkinsonian drugs (Holper et al., 2019). The receptors contributing to the drug effects to the greatest extent were adrenergic (α1B), dopaminergic (D1/2), glutaminergic (NMDA1, 3), histaminergic (H1), muscarinic (M1, 3), opioid (OP1-3), serotonergic (5-HT2A, 5-HT2C, 5-HT3A), and sigma (*σ* 1) receptors (Holper et al., 2019). For instance, the sigma 1 receptor showed the strongest effect in increasing complex I activity, and a significant effect in increasing complex IV activity (Holper et al., 2019). The sigma 1 receptor plays an important role in regulating mitochondrial apoptosis, proliferation, and neuroprotection, but it also modulates inflammatory and immune responses (Szabo et al., 2014, 2016; Brimson et al., 2018). Recently, a study in rats has shown that serotonin, through the 5-HT2A receptor, increased mitochondrial biogenesis, mitochondrial respiratory capacity, oxidative phosphorylation efficiency, and therefore ATP generation in cortical neurons (Fanibunda et al., 2019). Additionally, it exhibited a neuroprotective effect (Fanibunda et al., 2019). These effects take place through recruitment of modulators of mitochondrial biogenesis such as sirtuin SIRT1 and the transcriptional coactivator PGC-1a, which are also implicated in metabolic control and longevity (Fanibunda and Vaidya, 2021). It would therefore be interesting for future research to test whether 5HT or sigma 1 agonists alleviate fatigue in different conditions such as CFS, depression or MS through their effects on mitochondria and inflammatory activity.

Finally, oxygen-ozone autohemotherapy has been found to greatly improve fatigue symptoms in CFS patients (Tirelli et al., 2021). While the mechanisms through which ozone can impact fatigue are still unclear, it has been hypothesized that the antioxidant and immunomodulatory properties of ozone my play a role (Tirelli et al., 2021). Medical ozone effects include improved oxygen release by the red blood cells, immunomodulation, and regulation of cellular antioxidants (Viebahn-Haensler and León Fernández, 2021).

## Summary and Future Directions

We have presented theoretical and methodological considerations for defining sensations of fatigue and energy, distinguishing between subjective and objective measures, between trait, prolonged state, and state, as well as between different domains of energy-related sensations including cognitive, physical, emotional, motivational, social, and sexual. We also suggested that the speed through which energy-related sensations arise and dissipate might constitute a further dimension of interest that is currently poorly understood, which we term subjective energizability and fatigability, respectively. We have also defined sensations of energy as “X sensation of y energy availability related to a task demand,” with X representing state, prolonged state and trait and Y representing the six energy-related domains. While there is robust evidence that sensations of energy and fatigue are two separate constructs, we argue that the acquiescence effect needs to be addressed for conclusions.

A large body of evidence reveals a robust link between fatigue and different components of energy metabolism. These components include mitochondria structure, mitochondrial function, mitochondrial energy metabolism, immune response to mitochondria, the balance between sympathetic and parasympathetic activity, O_2_, and inflammation. However, the relation of energy metabolism with sensations of energy are poorly investigated.

Potential treatment options to improve bioenergetic dysregulation are still in its infancy but might consist of physical exercise, mitoprotective diets, supplementations addressing nutrient deficiencies (such as vitamin D or lower levels of enzymes such as CoQ10), and oxygen-ozone autohemotherapy. Further treatments might comprise psychotropic medications capable of improving mitochondrial bioenergetics, speculatively through 5HT or sigma 1 receptor actions.

**Figure 3:**
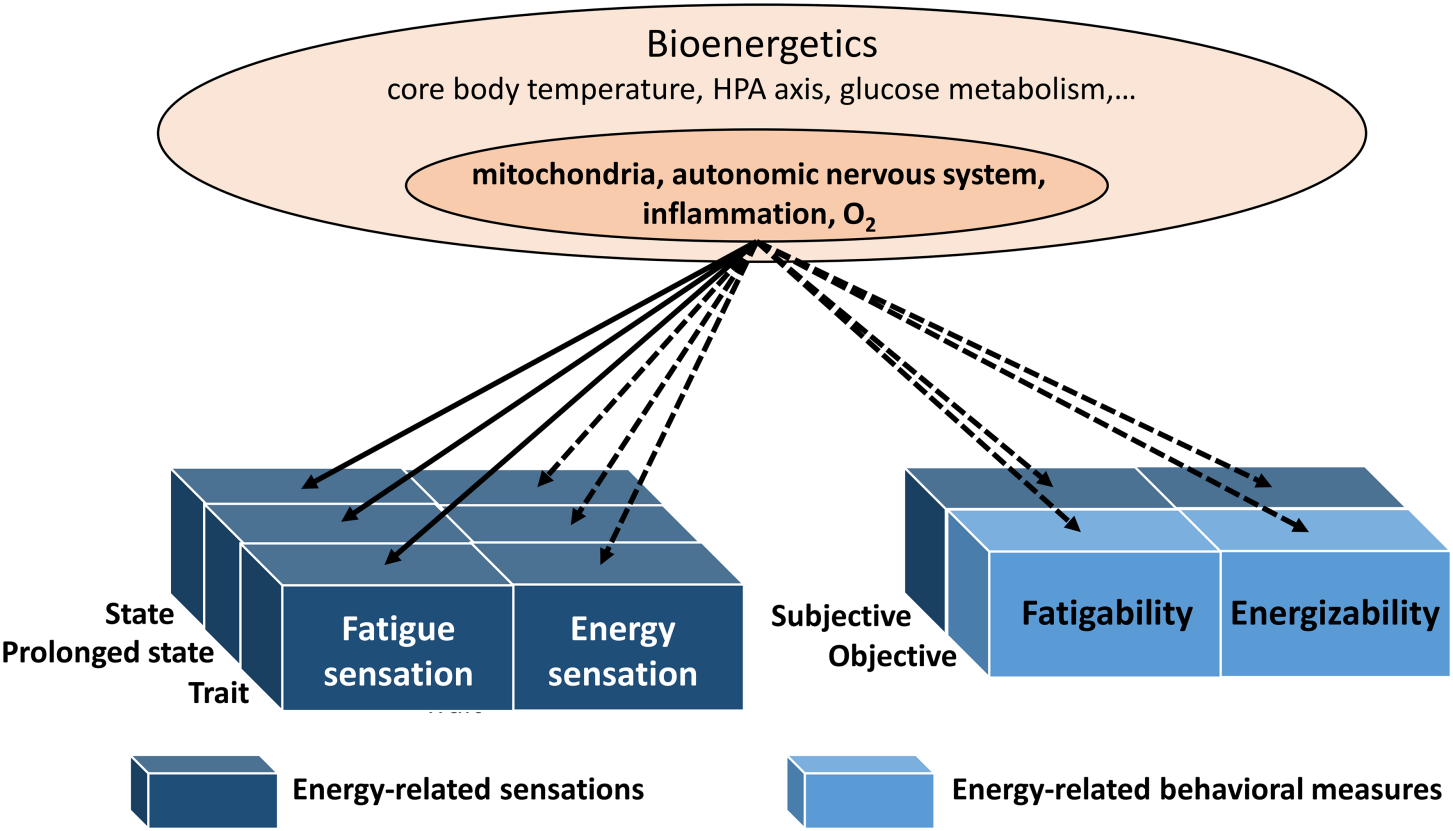
The bioenergetics components and their link with fatigue sensations, energy sensations, energizability, and fatigability. Solid lines: reviewed here. Dashed lines: not reviewed here. All these bioenergetic components may be linked to behavioral markers (see objective fatigability) and sensations (see subjective fatigability; fatigue and energy sensations). The concept of energizability has been newly introduced and subjective fatigability has been neglected.

Hypotheses for future research could address the temporal elements of energy-related sensations, something which has been neglected up to this point (see Figure 3). Therefore, we have introduced the concepts of subjective energizability and fatigability. The validity of these concepts and their biological underpinning could represent future research questions, allowing a better understanding of subjective and bioenergetics.

Finally, measures of intelligence (g factor) and psychopathology (p factor) have been found to covary (Fissler et al., 2022). For example, Caspi et al. (2014) found that individuals with higher psychopathology scored lower on a wide range of intelligence tests and showed more cognitive problems in everyday life as reported by informants who knew them well. In addition, both cognitive disorders (disorders of g) such as Alzheimer’s dementia and psychological disorders (disorders of p) such as depression show high comorbidity. The shared variation between measures of p and g, was defined as the cerebral function factor (c factor). Mitochondrial bioenergetic dysregulation has been proposed to be the biological underpinning of the c factor, or in other words, of the covariation between g and p (Fissler et al., 2022). As mitochondrial bioenergetics underlie energy-related sensations, the latter might provide a subjective indicator of the c factor (Fissler et al., 2022). Future research needs to test this hypothesis and reveal this possible link between energy-related sensations, the p factor, g factor and the c factor. If this hypothesis is verified, energy-related sensations may provide an easy-to-obtain marker of general cerebral function.

## Author Contributions

MF wrote the first draft of the manuscript. PF and RK conceptualized, edited, and revised the manuscript in a critical manner. All authors contributed to the article and approved the submitted version.

## Conflict of Interest

The authors declare that the research was conducted in the absence of any commercial or financial relationships that could be construed as a potential conflict of interest.

## Publisher’s Note

All claims expressed in this article are solely those of the authors and do not necessarily represent those of their affiliated organizations, or those of the publisher, the editors and the reviewers. Any product that may be evaluated in this article, or claim that may be made by its manufacturer, is not guaranteed or endorsed by the publisher.
